# Wenhua Juanbi Recipe Attenuates Rheumatoid Arthritis via Inhibiting miRNA-146a-Mediated Autophagy

**DOI:** 10.1155/2022/1768052

**Published:** 2022-11-16

**Authors:** Haili Zhou, Liuyun Huang, Kuijun Zhan, Xide Liu

**Affiliations:** ^1^Second Clinical College, Zhejiang Chinese Medicine University, Hangzhou, Zhejiang 310000, China; ^2^Department of Arthropathy, Zhejiang Hospital of Integrated Traditional Chinese and Western Medicine, Hangzhou, Zhejiang 310000, China

## Abstract

**Background:**

Wenhua Juanbi Recipe (WJR) is widely used for the treatment of rheumatoid arthritis (RA) in China. However, its mechanism of action remains unclear. This study was designed to investigate the potential therapeutic effects of WJR on the proliferation and apoptosis of synovial fibroblasts in RA and its efficacy in inhibiting miRNA-146a-mediated cellular autophagy.

**Methods:**

A collagen-induced arthritis (CIA) Wistar rat model was established. The model rats were administered WJR or methotrexate (MTX) to assess the therapeutic effect of the drugs. The chemical components of WJR were analyzed using UPLC-Q/TOF-MS. Histological changes; miRNA-146a, ATG5, ATG7, ATG12, Beclin1, LC3II, Bax, and Bcl2 expression; synovial apoptosis; and cellular proliferation were assessed. Primary synovial fibroblasts (FLS) were cultured *in vitro* using tissue block and transfected with miRNA-146a; an autophagy inducer was added to FLS, inhibiting the PI3K/AKT/mTOR pathway. FLS were cocultured with WJR-containing serum to observe the effects of miRNA-146a-mediated autophagy *via* the PI3K/AKT/mTOR pathway on CIA-affected rats.

**Results:**

Forty and thirty-one compounds were identified in WJR in the positive and negative ion modes, respectively. WJR significantly reduced toe swelling, arthritis scores, and expression of miRNA-146a and autophagy genes (ATG5, ATG7, ATG12, Beclin1, LC32, and Bcl2). Moreover, Bax expression, apoptosis, and attenuated proliferation were observed in rats. WJR could, therefore, regulate autophagy by influencing the miRNA-146a-mediated PI3K/AKT/mTOR pathway, which induces apoptosis and proliferation of FLS.

**Conclusion:**

WJR can inhibit autophagy, apoptosis, and proliferation in a CIA rat model by inhibiting the miRNA-146a-mediated PI3K/AKT/mTOR pathway.

## 1. Introduction

Rheumatoid arthritis (RA) is a refractory, systemic autoimmune disease affecting the joint synovial tissue, tendon sheaths, and bursa. It presents with joint inflammation and destruction, functional disability, and sleep disturbances, affecting both work and the quality of life [1–3]. Up to 1% of the world's population suffers from RA, with the highest incidence rates observed in women over 65 years of age [4]. Studies have reported that the progression of RA could be influenced by genetic, hormonal, and environmental factors [5]. Moreover, it has also been reported that treatment with antirheumatic drugs is not always effective, and the >development of drug resistance is common [6, 7]. Therefore, further research is needed to explore the pathogenesis of RA in order to develop novel and effective treatments.

It has been observed that RA patients experience abnormal miRNA regulation, leading to enhanced inflammatory pathway signaling and increased secretion of proinflammatory cytokines, which have the potential to induce tumorigenesis and autoimmune diseases [8]. miRNA-146a is directly or indirectly involved in the intrinsic and adaptive immune responses. Studies have demonstrated that RA could be caused by genetic polymorphisms of miRNA-146, especially in females [9]. Moreover, miRNA-146a may hamper MC3T3-E1 proliferation and induce apoptosis in mouse chondrocytes by controlling Bcl2 expression [10]. Autophagy is an important physiological process aiding the cellular response to injury by destroying unused components. During cellular stress, autophagy degrades intracellular components to produce adenosine triphosphate (ATP) and maintain essential cellular functions [11]. However, autophagy is a double-edged sword as it can trigger autoimmune diseases [12]. Autophagy promotes the development of RA by increasing the level of synovial tissues [13]. The mTOR complex 1 (mTORC1), a cellular energy level sensor, induces autophagy *via* nutrient starvation-induced inhibition. RA patients display an abnormal PI3K/AKT/mTOR signaling axis; therefore, inhibition of mTOR signaling can be a target for RA treatment [14, 15].

Currently, methotrexate, or other targeted synthetic antirheumatic drugs, is widely used for the treatment of RA [16]. However, these drugs are mostly either slow-acting or cause adverse effects after long-term treatment. Traditional Chinese medicine (TCM) can effectively treat chronic diseases and has been recognized as an alternative approach to treating RA [17, 18]. The Wenhua Juanbi Recipe (WJR) consists of Clematidis Radix, *Lonicera japonica*, Quan Scorpion, Centipede, Angelicae Dahuricae Radix, Stiff Silkworm, White Mustard Seed, Angelicae Dahuricae Radix, Bark of Erythrina variegata Linn, Rhizoma corydalis, and Coix Seed. Clematidis Radix can reduce arthritic symptoms in rats with collagen-induced arthritis (CIA) [19]. Similarly, *Lonicera japonica* can prevent bone erosion in the joints of a CIA rat model [20]. Centipede is clinically effective in treating RA when combined with other traditional Chinese medicines [21], while Coix lacryma has been shown to inhibit proinflammatory factors and reduce oxidative stress, thus preventing RA development [22]. Moreover, WJR may reduce miRNA-146a levels and improve inflammation in the CIA rat model [23]; however, its mechanism of action remains unclear. We, therefore, performed this study to investigate the therapeutic effects of WJR in a CIA rat model and explored possible mechanisms involved in treating RA utilizing *in vivo* and *in vitro* experiments.

## 2. Materials and Methods

### 2.1. Preparation and Identification of WJR

WJR, obtained from the Zhejiang Integrated Hospital of Traditional Chinese and Western Medicine (Hangzhou, China), consisted of Weilingxian (30 g), Rendongteng (20 g), Quanxie (6 g), Wugong (6 g), Fangfeng (10 g), Jiangcan (10 g), Baijiezi (10 g), Baizhi (10 g), Haitongpi (20 g), Yanhusuo (20 g), and Yiyiren (30 g). The phytochemicals available in the WJR were analyzed using UPLC-Q/TOF-MS technology. Specifically, the analyses were conducted using Waters ACQUITY I-Class Plus UPLC (Waters, USA) and SCIEX X-500R Q/TOF-MS (AB SCIEX, USA) systems. The operation was conducted in positive and negative ionization modes to obtain details related to relative molecular mass and tandem mass spectrometry of the relevant compounds. Data were interpreted using UNIFI software (Waters, USA, 2021).

### 2.2. CIA Rat Model

Female Wistar rats SPF class, weighing between 110 ± 10 g and 200 ± 20 g, were obtained from the Animal Experiment Center at the Zhejiang University of Traditional Chinese Medicine (ethics approval number 20200928-08). Rats were housed under constant temperature (22 ± 2°C), humidity (55 ± 10%), and light-dark cycle (07:00–19:00) conditions with free access to drinking water and food. After one week of adaptation to the laboratory conditions, Wistar rats were randomly divided into the control (*n* = 25) and experimental (*n* = 75) groups. Emulsified collagen (Chondrex Corporation, lot number: 2002–2) was mixed with incomplete adjuvant (Chondrex Corporation, lot number: 7002) at a 1 : 1 ratio to create a 2 mg/mL BC II emulsion. The emulsion was injected into the tail root of rats in the experiment group. The initial administration was 0.3 mL, followed by 0.15 mL after 7 d. The rats exhibited inflammation in the ankle and toe, redness and swelling in the paw, and a toe volume greater than 1.6 mL. AI scores were considered as follows: 0 = inflammation, 4 = heavy inflammation in the limbs and joints, and greater than 4 = successful establishment of the CIA model. After the model was successfully established, the rats were further randomly divided into three treatment groups: model (saline), WJR (intragastric administered with WJR at 22.9 g/kg/d), and MTX (intragastrically administered with MTX at 0.78 mg/kg/w) [24]. Animals were treated for four weeks.

### 2.3. Hematoxylin-Eosin Staining (H&E)

The rat synovial tissue was removed using scissors and washed in PBS (Zhejiang Gino Biomedical Technology Corporation, lot number: 1901050101) and then dried on filter paper. The dried samples were soaked in 4% paraformaldehyde (Shanghai Shenggong Bioengineering Corporation, lot number: E672002), paraffin-embedded, and sliced into 5 *μ*m sections before being baked for 1 h. After dewaxing, the samples were stained with hematoxylin at 37°C for 5 min, rinsed with water for 10 min then soaked in 1% hydrochloric acid for 1–5 s, rinsed with water for 10 min, and finally washed in distilled water for 1–2 s. The sections were then restained with 5% eosin for 10 to 180 s; serially dehydrated in 70%, 80%, 90%, and 95% alcohol for 5 min; and then further dehydrated with 100% alcohol for 10 min (H&E staining kit, Beijing Soleibao Technology Corporation, lot number: G1120). Sections were dewaxed by rinsing with xylene for 10 min and fixed with neutral resin. After drying, the synovial tissue from each group was examined under a light microscope.

### 2.4. Immunohistochemistry

Ki67 expression in the synovial membranes of the four groups of rats was measured employing immunohistochemistry (Proteintech Group, item no.: 27309-1-AP). TUNEL (In Situ Cell Death Detection Kit, Roche, Switzerland, lot number: 11684817910) was used to measure synovial membrane apoptosis. The sections from each group were dried at 60°C overnight to prevent desquamation. They were then washed thrice with PBS for 5 min and incubated in 3.0 g/L of hydrogen peroxide-methanol solution at 37°C. Following this, the sections were left at room temperature for 45 min, and excess liquid was ejected. The primary antibody Ki67 (1 : 200) was added dropwise and incubated overnight at 4°C. Negative control was washed thrice with PBS for 5 min instead of the primary antibody. Biotinylated goat anti-mouse secondary antibody (1 : 500) was then added dropwise before incubation at 37°C for 1 h. DAB color development (Beijing Zhongshan Jinqiao Biotechnology Corporation, lot number: K216405E), dehydration, and clear neutral gum were used to seal the films. The samples were examined using a light microscope; five fields of view were selected for each section with positive (proliferating cells) seen as brown or tan and granular. The proliferation index was calculated as the percentage of proliferating cells in 10 slices for each group.

The 4% paraformaldehyde has repaired rat synovial tissue on the other side. Paraffin-embedded tissue sections were dewaxed and hydrated and rinsed twice with PBS, and a proteinase K working solution was added and reacted at 21–37°C for 15–30 min. The samples were rinsed twice with PBS for 5 min each, and the water contained in the samples was wiped off. TUNEL reaction mixture (50 *μ*L) was added dropwise to the samples and incubated for 1 h at 37°C in a wet box (a coverslip was used during incubation to prevent evaporation and maintain uniform distribution of the TUNEL reaction mixture). The samples were rinsed thrice with PBS for 5 min and now are ready for analysis under fluoroscopy. DAB substrate solution (50–100 *μ*L) was added before, and samples were incubated for 10 min at room temperature and then rinsed again thrice with PBS for 5 min. Hematoxylin restained cell nuclei, blocked slices, and image analysis were under light microscopy; significant tissue phases were selected and observed under the microscope.

### 2.5. Reverse Transcription-Quantitative Polymerase Chain Reaction (RT-qPCR)

Total RNA was extracted using the TRIzol method to determine its concentration; appropriate primers are shown in Table 1. The reaction system of miRNA-146a and common RNA reverse-transcribed cDNA was 15 *μ*L, according to the kit instructions (PrimeScript™ RT reagent Kit with gDNA Eraser, Takara, Japan, lot number: AHG1528A; All-in-One miRNA First-strand cDNA Synthesis Kit, GeneCopoeia, USA, lot number: B13WY0301). PCR cycles were performed under the following conditions: 37°C for 180 min and 85°C for 5 min, then 4°C. Real-time fluorescence qPCR reactions were carried out using a 20 *μ*L system for miRNA and a 15 *μ*L system for common genes. Reagents were added according to the kit instructions (FastStart Universal SYBR Green Master, Roche, Basel, Switzerland, lot number: 11610100). Forty cycles were performed based on the reaction conditions. U6 was chosen as the miRNA internal reference and GAPDH housekeeping genes as the ATG5, ATG7, and ATG12 internal references, and all reaction systems were performed in duplicate. The normalized 2^-*ΔΔ*Ct^ values were calculated to determine the expression contents of miRNA-146a, ATG5, ATG7, and ATG12. The experiments were performed in triplicate.

### 2.6. Western Blot

To extract and quantify the protein, cell lysate and protease inhibitor were added to 100 mg of milled synovial tissue. Protein samples (50 *μ*g) were added to 10% SDS-PAGE (Beijing Puli Gene Technology Corporation, lot number: 2019F3KB1005) for electrophoresis for 1.5 h, followed by transfer onto the PVDF membrane. Afterwards, the membrane was soaked in 5% skimmed milk for 2 h and incubated overnight at 4°C with the following primary antibodies: Beclin1 (Absin, item no.: abs131205), LC3II (CST, item no.: 3868), Bax (CST, item no.: 14796), Bcl2 (Proteintech, item no.: 60178-1-1 g), PI3K (CST, item no.: 4249), AKT (Immunoway, item no.: YM3618), and mTOR (CST, item no.: 2983), and washed thrice in TBST (Beijing Puli Gene Technology Corporation, lot number: 201902 EB1009) buffer for 10 min. HRP-labeled secondary antibody was added before incubation for 1 h at room temperature and washed thrice in TBST for 10 min, followed by soaking in ECL (Millipore Corporation, lot number: 1807102). The reaction mixture was placed in a fully automated chemical imaging system for the examination of protein expression. The experiments were performed in triplicate.

### 2.7. Culture and Identification of Synovial Fibroblasts (FLS)

The synovial membranes from the normal and CIA rat groups were moved to a culture dish on an ultraclean bench and washed in PBS containing 10% penicillin-streptomycin solution (Zhejiang Senrui Biotechnology Corporation, lot number: 2011100501). Synovial membranes were cut into 2–3 mm^3^ tissue blocks using sterile surgical scissors and placed into a 12-well culture plate containing 2 mL of 20% FBS (Gibco, lot number: 2305262RP) high-sugar type DMEM medium (Gibco, lot number: 2186872). The culture plate was placed in an incubator at 37°C with 5% CO_2_, and the plate was observed daily. Most of the cells from the tissue block were primary FLS, with few being macrophage-like cells, with a survival rate of 3–4 d. After approximately 14 d of cell culture, the old medium was discarded and 1 mL of PBS was added to each well, followed by 800 *μ*L of trypsin for digestion for 10 min. Serum was then added to stop the digestion process, and the samples were centrifuged at 1500 rpm for 5 min at 4°C. The cells were transferred into a 6 cm culture dish containing 4 mL of 10% FBS high-sugar type DMEM medium and incubated at 37°C with 5% CO_2_. Cells were cultured until the third generation was ready for experimental manipulation.

Since there is no distinct marker for FLS, Vimentin can identify whether the collected cells are fibroblast- or macrophage-like. In this study, Vimentin was used for cell identification. Sterilized slides were placed in a 24-well plate, an appropriate amount of medium was added, and air bubbles were removed. FLS (4 × 10^4^) cells were added to achieve a cell fusion rate of 50% after 24–36 h. The medium was discarded, and the cells were washed twice in PBS. The sample was soaked in 500 *μ*L of 4% paraformaldehyde for 30 min, washed thrice in PBS, combined with perforating solution, and left to stand for 30 min at room temperature. Afterwards, the sample was washed twice in PBS. A corresponding concentration of anti-Vimentin antibody was added to the sample and kept overnight at room temperature and then washed twice in PBS. The sample was soaked in 3% skimmed milk for 30 min at room temperature and washed twice with PBS. A secondary antibody was added, and the sample was left at 37°C for 30 min and washed with PBS twice, before adding DAPI solution (Shanghai Biyuntian Biotechnology Corporation, Item no.: P0131). The sample was left at room temperature for 5 min, washed PBS was added to wash the medium twice in PBS, and the specimen was sealed. The sample was examined using a fluorescence microscope (Carl Zeiss AG, model number: ZEISS axiovert 200).

### 2.8. Cell Grouping and Cell Counting Kit-8 (CCK-8) Assay

Cells (approximately 2,000) were distributed into each well of a 96-well plate. Medium (100 *μ*L) without double antibodies was added to each well before incubation for 24 h. CCK-8 solution (10 *μ*L) (Shanghai Biyuntian Biotechnology Corporation, Item no.: C0042) was added to the wells 1 h before examining the cells. A well with no cells but equal amounts of cell medium and CCK-8 solution was used as a blank control. The absorbance at 450 nm was measured on an enzyme marker.

### 2.9. Flow Cytometry

Each staining reagent was centrifuged, and the liquid from the wall and mouth of the tube was collected. Binding Buffer (4x) was diluted in distilled water to prepare 1x Buffer solution. The collected cells were rinsed with PBS, shaken gently, and resuspended in 195 *μ*L of 1x Binding Buffer to obtain a cell density of 2-5 × 10^5^/mL. Annexin V-FITC (5 *μ*L) (Shanghai Yishan Biotechnology Corporation, lot number: AP001) was added to resuspended cells (195 *μ*L) and mixed well and incubated at room temperature for 10–15 min. 5 *μ*L of Annexin V-FITC was added to 195 *μ*L of resuspended cells and incubated at room temperature for 10–15 min. Propidium iodide (10 *μ*L) was added and assayed within 4 h to avoid fluorescence decay.

### 2.10. Cell Transfection of miRNA-146a Mimic and Inhibitor

FLS were seeded into 24- or 6-well plates 24 h before transfection. miRNA mimic NC, miRNA-146a mimic, miRNA inhibitor NC, and miR-146a inhibitor (QIAGEN, lot number: 195044394) were cocultured with Lipofectamine 3000 (Invitrogen, lot number: 2067565) at a final concentration of 80 nM. The cells were harvested at 24 h and 48 h for RNA and protein extraction, respectively.

### 2.11. 3-MA, Rapamycin, and LY294002 Addition

Autophagy inhibitor (1%; 5 mM) 3-MA (MCE, HY-19312) was added [25], followed by 10 nM of 1% autophagy inducer rapamycin (SIGMA, B39500) and 20 *μ*M of 1% PI3K inhibitor LY294002 (MCE, HY-10108). A 1 mL cell culture medium was added containing 1 *μ*L each of the 3-MA, rapamycin, and LY294002 solutions.

### 2.12. Preparation of Drug-Containing Rat Serum

After one week of adaptive feeding, SD rats (male, SPF class, 4-5 weeks old) were obtained from the Animal Experiment Center at the Zhejiang University of Traditional Chinese Medicine (ethics approval number 20200928-08). SD rats were housed under constant temperature (22 ± 2°C), humidity (55 ± 10%), and light-dark cycle (07:00–19:00) conditions with free access to drinking water and food); the animals were treated with WJR (22.9 g/kg/d) twice daily for 3 d. After 1 h of treatment, the rats were anesthetized with 3% pentobarbital, and blood samples were obtained from their abdominal aortas and centrifuged at 3,000 rpm for 15 min. The supernatant was inactivated (56°C, 30 min) and filter-sterilized using a 0.22 *μ*m filtration membrane (Hangzhou Dawen Biological Corporation, lot number: 9002-93-1) and stored at −80°C for further studies.

### 2.13. Statistical Analysis

The experimental data were analyzed using SPSS 25.0 software (IBM Corp. in Armonk, NY, USA). Data are expressed as the average ± SD derived from three separate experiments in triplicates. One-way analysis of variance (ANOVA) was used to compare groups, Tukey's test was used to compare two means of multiple groups, and a paired *t*-test was used to compare within groups. All statistical tests were two-sided, with statistically significant differences set at *P* < 0.05.

## 3. Results

### 3.1. Determination of Chemical Components of WJR

**Figure 1:**
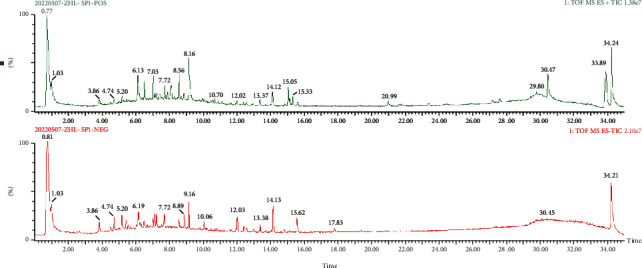
Characteristics of chemical components of WJR analyzed by UHPLC-Q-TOF/MS.

The chemical components of WJR were analyzed using UHPLC-Q/TOF-MS under positive and negative ion modes.  Figure 1 and Table 2 indicate that WJR contained 40 and 31 compounds in the positive and negative ion modes, respectively.

### 3.2. WJR Effectiveness in Treating CIA Rat Model

**Figure 2:**
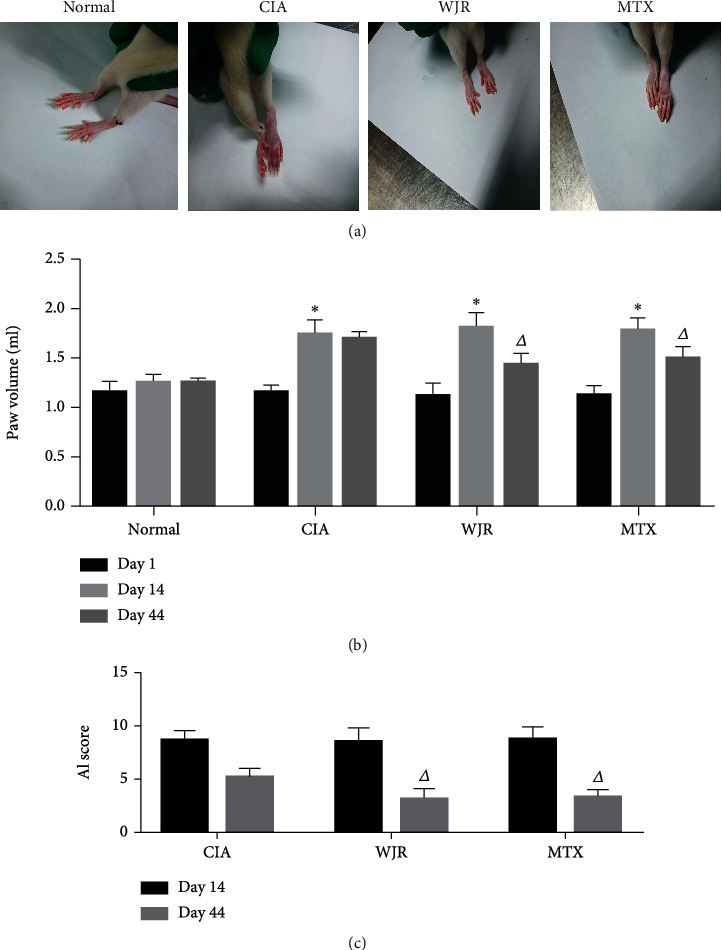
The model group exhibited significant toe swelling and high AI scores, while the WJR and MTX groups exhibited significantly less toe swelling and significantly lower AI scores (*n* = 10). (a) Appearance of the paws of rats in each group. (b) Paw volume of rats in each group. (c) AI score of rats in each group. Compared with those in the normal group in the same period, ^∗^*P* < 0.05; compared with the model group in the same period, ^△^*P* < 0.05.

After 14 d of establishing the model, rats in the experimental group exhibited swollen joints, redness at toe ends, extreme thickening of the toes, and elevated AI scores (Figure 2(a)). After 30 d of WJR treatment, toe swelling, and AI scores were significantly (*P* < 0.05) improved in the WJR and MTX groups (Figures 2(b) and 2(c)).

### 3.3. Histological Changes in Synovial Tissue

**Figure 3:**
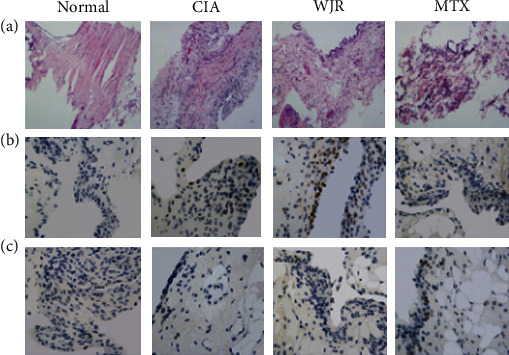
Both WJR and MTX can reduce proliferation and promote apoptosis: (a) results of H&E staining of synovial tissue of rats in each group (×100); (b) results of synovial tissue Ki67 analysis of rats in each group (magnification ×400); (c) results of TUNEL analysis of synovial tissue of rats in each group (magnification ×400).

The histological changes in the synovial tissues after H&E staining are shown in Figure 3(a). The synovial cells in the normal group displayed no hyperplasia. The rats in the CIA model group exhibited synovial cell proliferation in three layers, disorganized arrangement of cells, synovial tissue blockage and edema, capillary hyperplasia, inflammatory cell infiltration, and abundant intrasynovial vessels. In the WJR and MTX groups, the synovial tissue structure was significantly improved exhibiting reduced blockage and edema, mild hyperplasia in synovial cells, decreased number of infiltrating inflammatory cells, and inhibition of synovial fibrous tissue proliferation. Ki67 expression was significantly increased in the CIA rat group compared to animals in the normal counterpart (*P* < 0.05); on the other hand, its expression was significantly (*P* < 0.05) decreased in the WJR and MTX groups (Figure 3(b)). The apoptosis rate in the CIA rat group was significantly (*P* < 0.05) reduced, while it was significantly (*P* < 0.05) increased in the WJR and MTX groups (Figure 3(c)).

### 3.4. Effects of WJR on Expression of miRNA-146a and Autophagy-Related Genes and Autophagy and Apoptosis Proteins

**Figure 4:**
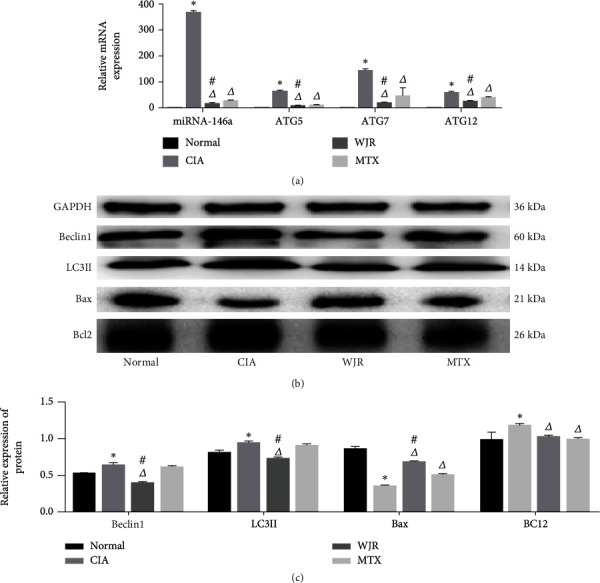
WJR treatment reduced the expression of miRNA-146a, autophagy-related mRNA, and proteins, increased the expression of the proapoptotic gene (Bax), and inhibited the expression of the inhibitory apoptotic gene (Bcl2) in synovial tissues, to a greater extent than that in the MTX group. The differences were statistically significant (*P* < 0.05): (a) expression of miRNA-146a, ATG5, ATG7, and ATG12 in the synovial tissues of each group; (b, c) expression of Beclin1, LC3II, Bax, and Bcl2. Compared with the normal group, ^∗^*P* < 0.05; compared with the CIA group, ^△^*P* < 0.05; compared with the MTX group, ^#^*P* < 0.05.

RT-qPCR and Western blot were employed to identify the expression of miRNA-146a, autophagy-related genes, and apoptotic proteins in synovial tissues. The expression of miRNA-146a, ATG5, ATG7, and ATG12 in the CIA rat group was significantly (*P* < 0.05) increased, whereas that of autophagy-related genes was significantly (*P* < 0.05) reduced in the WJR and MTX rat groups (Figure 4(a)). Beclin1, LC3II, and Bcl2 protein expression was increased, while that of Bax protein was decreased in the CIA rat group (*P* < 0.05). Beclin1, LC3II, and Bcl2 protein expression was significantly decreased, and Bax protein expression was significantly increased in the WJR rat group (*P* < 0.05). Bax protein expression in the MTX rat group was significantly increased, whereas that in Bcl2 was significantly reduced (*P* < 0.05). Conversely, rats in the WJR group exhibited a significant reduction in Beclin1 and LC3II protein expression and an increase in Bax protein expression (*P* < 0.05; Figures 4(b) and 4(c)).

### 3.5. Vimentin Expression in FLS

**Figure 5:**
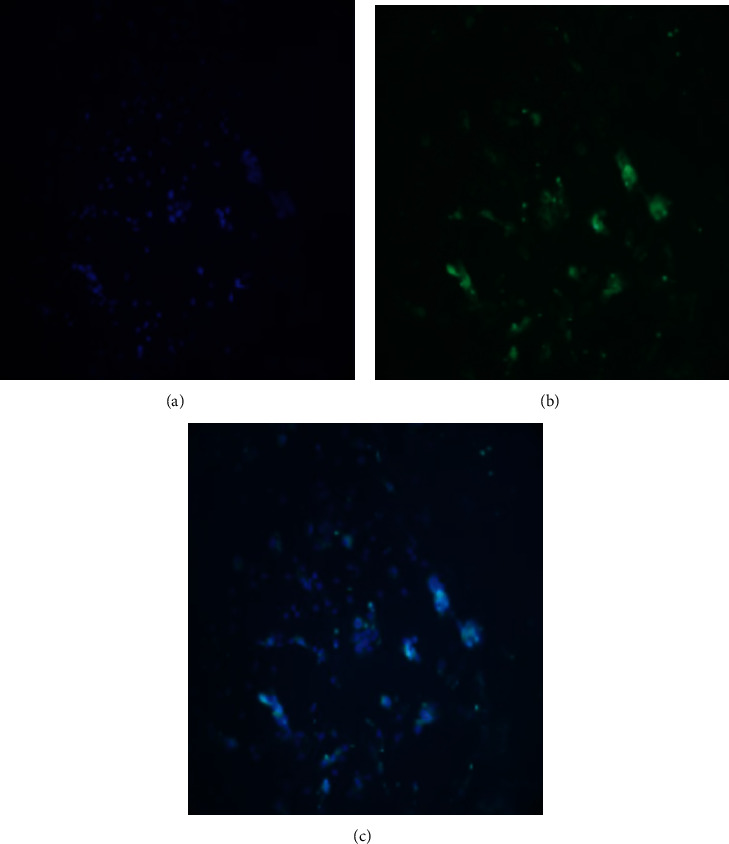
Vimentin is positively expressed in FLS (magnification ×100): (a) nucleus labeled with DAPI; (b) cell membrane labeled with Vimentin; (c) (a) and (b) merged.

FLS are mainly located in the synovial tissue along with a small number of MLC, fibroblasts, and vascular endothelial cells, as well as other cells. Fibroblasts can be identified by the presence of Vimentin protein, which is abundant in these cells. Using immunofluorescence, 90% of FLS cells obtained were positive for Vimentin on the cell membrane, and the purity of cells identified after merging was above 90% (Figure 5).

### 3.6. Effects of miRNA-146a on Autophagy, Apoptosis, and FLS Proliferation

**Figure 6:**
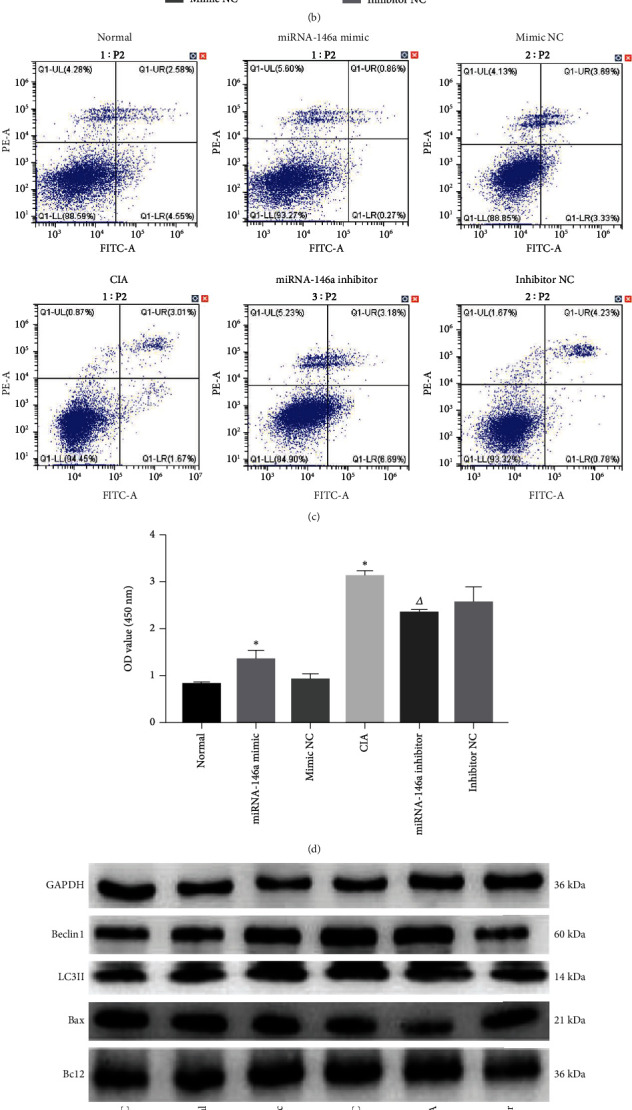
Effects of miRNA-146a on autophagy, apoptosis, and proliferation of FLS. (a) Expression of miRNA-146a was detected by RT-PCR after transfection of miRNA-146a or miRNA-146a inhibitor. (b) mRNA expression of ATG5, ATG7, and ATG12. (c) Apoptosis rate in each group. (d) OD values in each group. (e–g) Protein expressions of Beclin1, LC3II, Bax, and Bcl2. Compared with the normal group, ^∗^*P* < 0.05; compared with the CIA group, ^△^*P* < 0.05.

Transfection of miRNA-146a mimic increased miRNA-146a expression, while transfection of miRNA-146a inhibitor significantly reduced miRNA-146a expression (Figure 6(a)). RT-qPCR showed that the expression of ATG5, ATG7, and ATG12 was significantly (*P* < 0.05) increased in both, normal transfected miRNA-146a and CIA rat groups. The expression of ATG5, ATG7, and ATG12 was significantly (*P* < 0.05) decreased in the CIA antisense-transfected miRNA-146a rat group (Figure 6(b)). Flow cytometry showed that the apoptosis rate was greatly decreased (*P* < 0.05) in the normal transfected miRNA-146a and CIA rat groups. However, the rate of apoptosis was significantly (*P* < 0.05) increased in the CIA antisense-transfected miRNA-146a animal group (Figure 6(c)). CCK-8 showed that the OD values of cells in the normal transfected miRNA-146a and CIA groups were significantly increased, whereas those in the CIA antisense-transfected miRNA-146a animal group were significantly (*P* < 0.05) decreased (Figure 6(d)). Western blot results indicated a significant increase in Beclin1, LC3II, and Bcl2 expression and decreased Bax counterpart in the normal transfected miRNA-146a and CIA rat groups (*P* < 0.05), whereas the expression of the former three proteins was reduced and the latter protein was increased in the CIA antisense-transfected miRNA-146a rat group (*P* < 0.05) (Figures 6(e)–6(g)).

### 3.7. Effects of Autophagy on Apoptosis and FLS Proliferation

**Figure 7:**
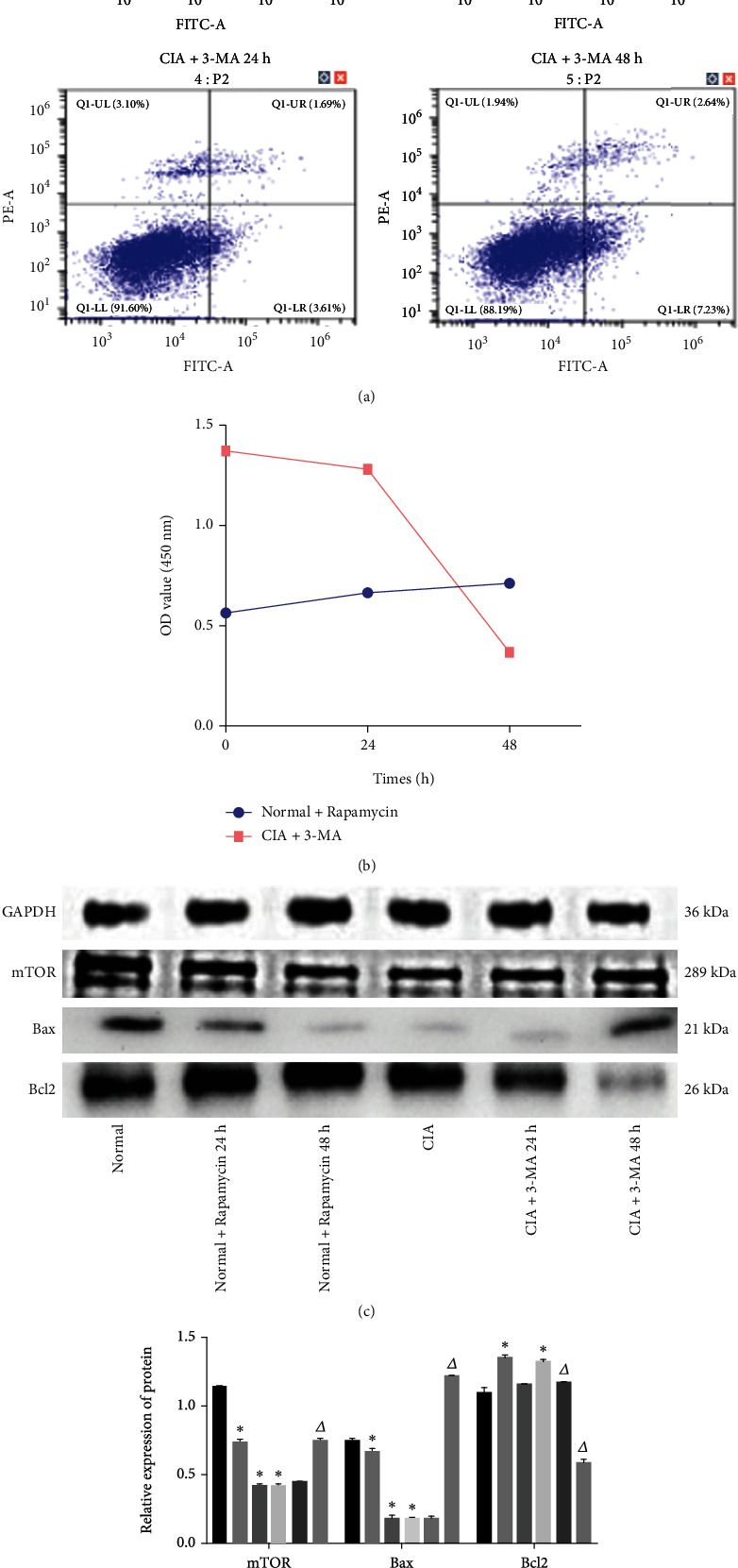
Effects of autophagy on FLS apoptosis and proliferation with autophagy inhibitors and agonists acting on FLS: (a) apoptosis rate in each group; (b) OD values in normal and CIA groups; (c, d) protein expressions of mTOR, Bax, and Bcl2. Compared with the normal group, ^∗^*P* < 0.05; compared with the CIA group, ^△^*P* < 0.05.

Flow cytometry was used to assess apoptosis, which was significantly decreased in rats in the normal and CIA groups after rapamycin treatment for 24 and 48 h. The apoptosis rate was significantly (*P* < 0.05) increased in the CIA rat model after treatment with 3-MA for 24 and 48 h (*P* < 0.05; Figure 7(a)). CCK-8 analysis showed that the OD values of cells in the normal and CIA rat groups after rapamycin treatment for 24 and 48 h were significantly increased, while those in the CIA rat model treated with 3-MA for 24 and 48 h were significantly reduced (*P* < 0.05) (Figure 7(b)). Western blot results demonstrated that cellular mTOR and Bax protein expression was significantly decreased in the normal and CIA animal groups after rapamycin treatment for 24 and 48 h, and Bcl2 protein expression was significantly increased after treatment for 24 h (*P* < 0.05). Cellular mTOR and Bax protein expression was significantly increased in the CIA rat model treated with 3-MA for 48 h, and Bcl2 protein expression was significantly (*P* < 0.05) decreased after treatment for 24 and 48 h (Figures 7(c) and 7(d)).

### 3.8. Effect of miRNA-146a on Apoptosis and PI3K/AKT/mTOR Pathway-Mediated FLS Proliferation

**Figure 8:**
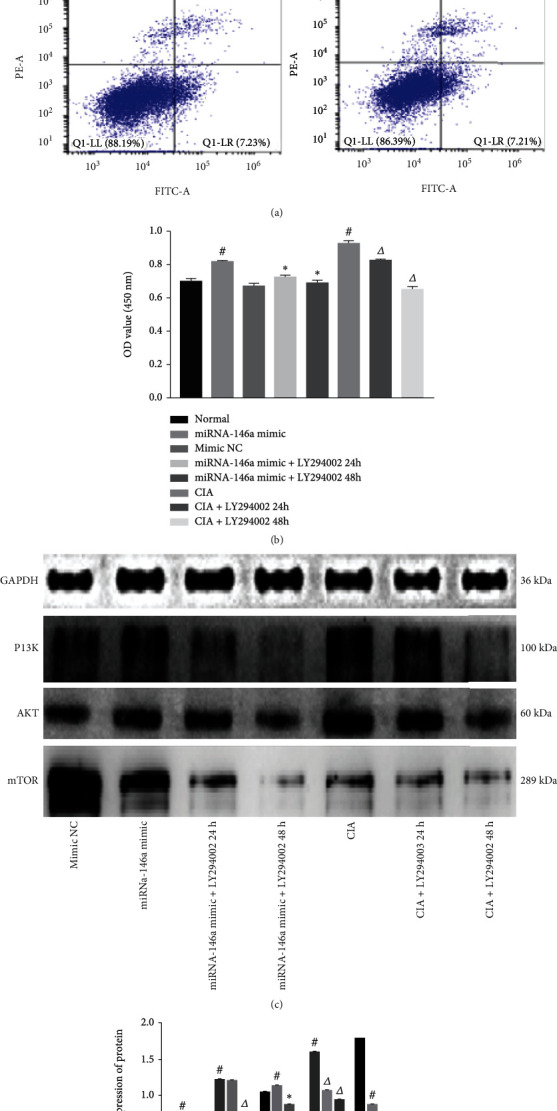
miRNA-146a regulates autophagy through the PI3K/AKT/mTOR pathway, resulting in decreased FLS apoptosis and increased proliferation: (a) apoptosis rate in each group; (b) OD values in each group; (c, d) protein expressions of PI3K, AKT, and mTOR. Compared with the normal transfected miRNA-146a NC group, ^#^*P* < 0.05; compared with the normal transfected miRNA-146a group, ^∗^*P* < 0.05; compared with the CIA group, ^△^*P* < 0.05.

Flow cytometry revealed that the rate of apoptosis was decreased in the normal transfected miRNA-146a and CIA rat groups, whereas the rate increased in the normal transfected miRNA-146a rat group treated with LY294002 for 24 and 48 h (*P* < 0.05). The CIA rat model exhibited a significant (*P* < 0.05) increase in the apoptosis rate after treatment with LY294002 for 24 and 48 h (Figure 8(a)). CCK-8 analysis showed that the OD values of cells in the normal transfected miRNA-146a and CIA groups were significantly increased; the OD values of cells in the normal transfected miRNA-146a group treated with LY294002 for 24 and 48 h were significantly reduced. The OD values of CIA model cells treated with LY294002 for 24 and 48 h were moderately reduced (*P* < 0.05; Figure 8(b)). Western blot results showed that rats in the normal transfected miRNA-146a and CIA groups exhibited an increased PI3K and AKT protein expression and decreased mTOR counterpart (*P* < 0.05); the expression of PI3K, AKT, and mTOR was reduced in the normal transfected miRNA-146a rat group treated with LY294002 for 24 and 48 h (*P* < 0.05). PI3K, AKT, and mTOR protein expression was reduced in rats in the CIA group when treated with LY294002 for 48 h (*P* < 0.05) (Figures 8(c) and 8(d)).

### 3.9. Effect of WJR on PI3K/AKT/mTOR Pathway-Mediated Protein Expression

**Figure 9:**
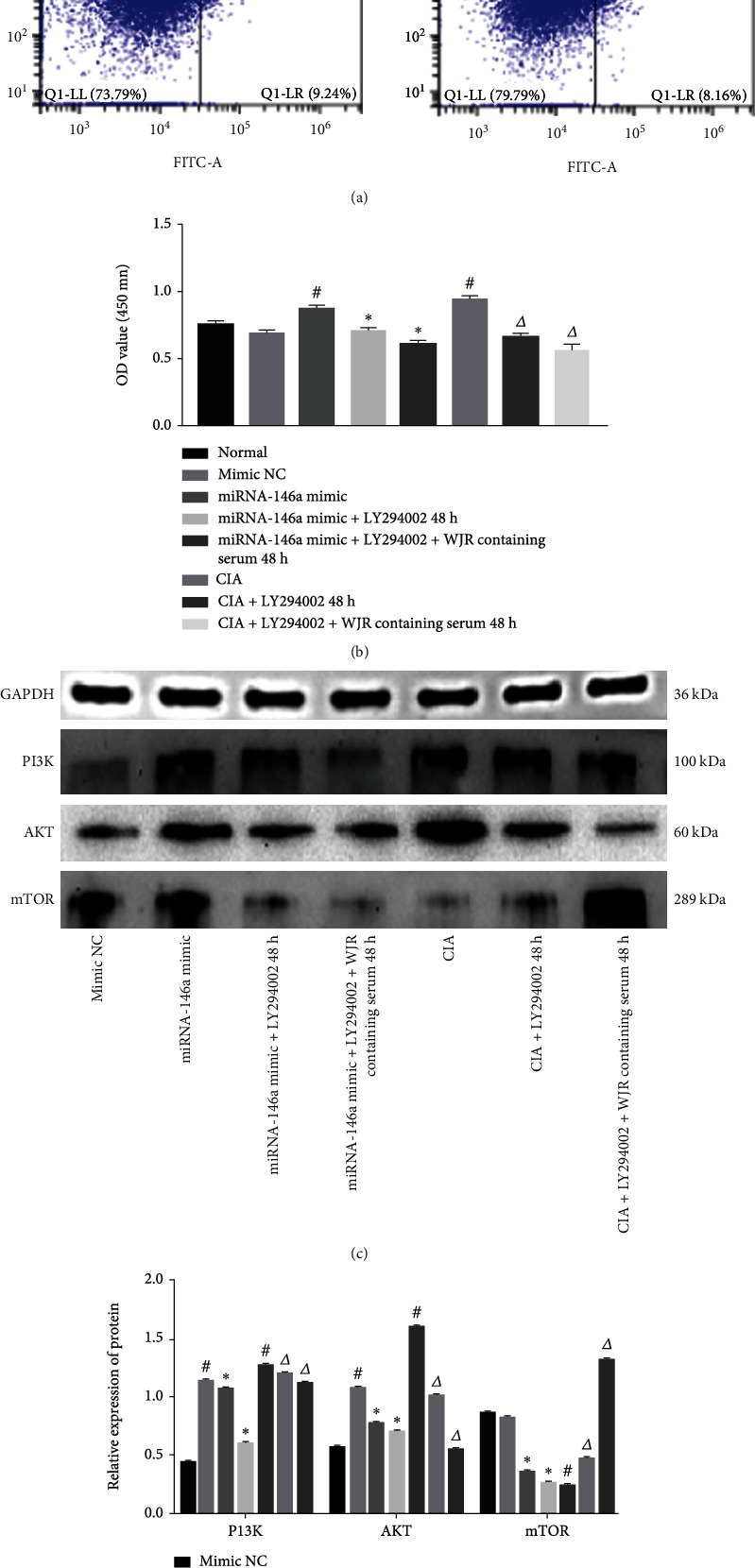
WJR increased FLS apoptosis, decreased proliferation, and decreased PI3K/AKT signaling in the normally transfected miRNA-146a and CIA groups: (a) apoptosis rate in each group; (b) OD values in each group; (c, d) protein expressions of PI3K, AKT, and mTOR. Compared with the normal transfected miRNA-146a NC group, ^#^*P* < 0.05; compared with the normal transfected miRNA-146a group, ^∗^*P* < 0.05; compared with the CIA group, ^△^*P* < 0.05.

Flow cytometry analysis showed that the rate of apoptosis was reduced (*P* < 0.05) in the normal transfected miRNA-146a and CIA rat groups; the rate was increased in the normal transfected miRNA-146a animal group treated with LY294002, as well as in the same treated group, but plus WJR-containing serum. Apoptosis rates increased in the CIA rat group treated with LY294002 and in the CIA group treated with LY294002 plus WJR-containing serum (*P* < 0.05) (Figure 9(a)). CCK-8 analysis showed that the OD values of cells in the normal transfected miRNA-146a and CIA groups were increased; the OD values of cells in the normally transfected miRNA-146a group treated with LY294002 and normally transfected miRNA-146a group treated with LY294002 plus WJR-containing serum were reduced. OD values of the CIA rat model cells treated with LY294002 and CIA rat model cells treated with LY294002 plus WJR-containing serum were reduced (*P* < 0.05) (Figure 9(b)). Western blot results showed that rats in the normal transfected miRNA-146a group revealed elevated PI3K and AKT protein expression, and those in the CIA group had elevated PI3K and AKT and lowered expression of mTOR protein; rats in the normal transfected miRNA-146a group treated with LY294002 and animals in the normal transfected miRNA-146 group treated with LY294002 plus WJR-containing serum displayed lower PI3K, AKT, and mTOR protein expression. PI3K and AKT protein expression was decreased, and mTOR protein expression was increased in the CIA rat model treated with LY294002 and the CIA rat model treated with LY294002 plus WJR-containing serum (*P* < 0.05) (Figures 9(c) and 9(d)).

### 3.10. Effects of WJR on miRNA-146a-Mediated FLS Autophagy

**Figure 10:**
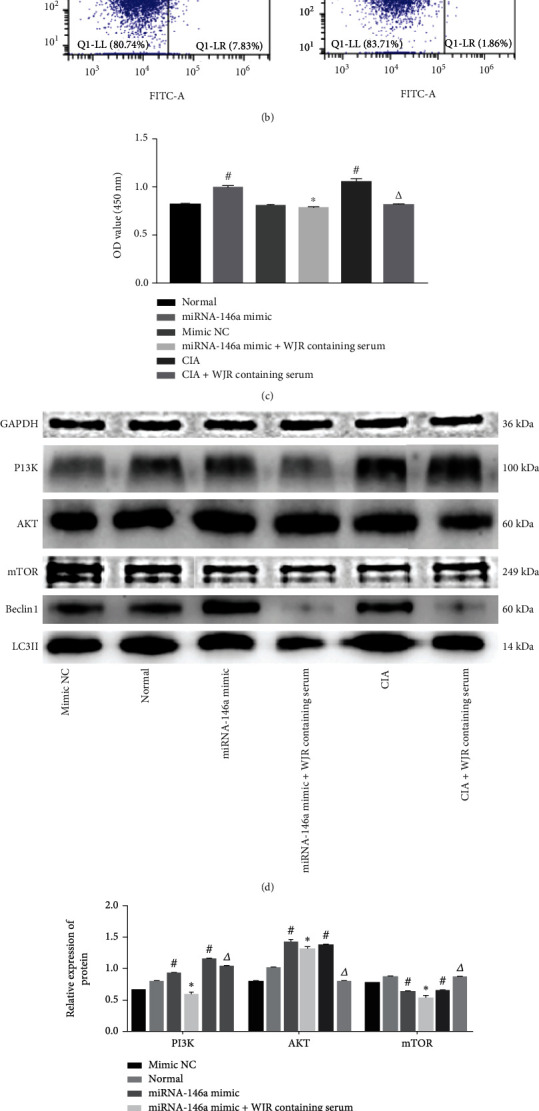
WJR decreased autophagy and proliferation and promoted apoptosis in the normal transfected miRNA-46a and CIA groups: (a) expression of miRNA-146a, ATG5, ATG7, and ATG12; (b) apoptosis rates in each group; (c) OD values in each group; (d–f) expression of PI3K, AKT, mTOR, Beclin1, and LC3II. Compared with the normal group, ^#^*P* < 0.05; compared with the normal transfected miRNA-146a group, ^∗^*P* < 0.05; compared with the CIA group, ^△^*P* < 0.05.

The expression of miRNA-146a, ATG5, ATG7, and ATG12 in the normal transfected miRNA-146a and CIA rat groups was significantly increased; the expression of miRNA-146a, ATG5, ATG7, and ATG12 was decreased in the normal transfected miRNA-146a animal group treated with WJR-containing serum. The expressions of miRNA-146a, ATG5, ATG7, and ATG12 were reduced in the CIA rat model treated with WJR-containing serum (*P* < 0.05) (Figure 10(a)). Flow cytometry studies showed that apoptosis rates were reduced in the normal transfected miRNA-146a and CIA rat groups; the rates were increased in rats in the normal transfected miRNA-146a group treated with WJR-containing serum. Apoptosis rates in the CIA rat model treated with WJR-containing serum were increased (*P* < 0.05) (Figure 10(b)). CCK-8 analysis showed that the OD values of cells in the normally transfected miRNA-146a and CIA rat groups were increased; the OD values of cells in the normally transfected miRNA-146a rat group treated with WJR-containing serum were reduced. The OD values of cells in the CIA rat model treated with WJR-containing drug serum were significantly decreased (*P* < 0.05) (Figure 10(c)). Western blot results revealed that the expression of PI3K, AKT, Beclin1, and LC3II was increased, and that of mTOR was decreased in the normally transfected miRNA-146a and CIA rat groups; the expression of PI3K, AKT, mTOR, Beclin1, and LC3II was reduced in the normal transfected miRNA-146a rat group treated with WJR-containing serum. The expression of PI3K, AKT, Beclin1, and LC3II was decreased, while that of mTOR was increased, in CIA-affected rats treated with WJR-containing serum (*P* < 0.05) (Figures 10(d)–10(f)).

## 4. Discussion

WJR displays anti-inflammatory, improved bone erosion, and reduced oxidative stress properties. Based on our UPLC-Q/TOF-MS analysis, 64 compounds were identified in WJR employing the positive and negative ion modes, all of which play important roles in pyretolysis, analgesia, anti-inflammation, and immune regulation. This study investigated the role of WJR-induced miRNA-146a in regulating FLS proliferation and apoptosis *via* the regulation of autophagy and highlighted the potential use of WJR as a therapeutic agent for treating RA. We noted that treatment of the rat model with WJR resulted in a significant reduction in toe swelling, arthritis severity, expression of miRNA-146a, and expression of autophagy genes (ATG5, ATG7, ATG12, Beclin1, LC32, and Bcl2). Moreover, autophagy may be regulated by WJR by influencing the miRNA-146-mediated PI3K/AKT/mTOR pathway, which could induce apoptosis and the proliferation of FLS.

Visual observations, AI scores, and H&E staining indicated the incidence of swelling and inflammatory infiltration of synovial tissues in the CIA rat model. We believe that the administration of WJR could ease these symptoms. Moreover, we noted that WJR reduced cell proliferation, thus promoting apoptosis of synovial tissues in the CIA rat model, as assessed by Ki67 and TUNEL methods. Furthermore, WJR treatment can reduce miRNA-146a levels and autophagy expression in a CIA rat model, thus providing potential mechanisms for promoting apoptosis and inhibiting FLS proliferation. In addition, miRNA-146a could inhibit FLS proliferation, promote apoptosis, and suppress autophagy, all of which are consistent with prior studies [26]. Finally, the increased expression of miR-146a was significantly correlated with the activation of inflammatory pathways in RA. The admiration of WJR could reduce miRNA-146a levels, suggesting its role in the treatment of RA.

Autophagy is a highly conserved process of material recycling in eukaryotic cells, and its activation is associated with RA-FLS resistance. Increased levels of apoptosis and decreased levels of proliferation were observed after the inhibition of autophagy in the CIA rat model [27]. Conversely, inhibition of autophagy renders cells more sensitive to apoptosis, and it is this balance that is disrupted during RA progression. High levels of autophagy increase apoptosis resistance in FLS cells, speeding up RA progression and causing severe abnormalities in autophagy and apoptosis. Therefore, it is crucial to maintain a balanced state between autophagy and apoptosis. After administration of WJR-containing serum, the expression of FLS autophagic genes and proteins (ATG5, ATG7, ATG12, Beclin1, and LC3II) in normal rats transfected with miRNA-46a was decreased, expression of the proapoptotic protein Bax was decreased, and that of antiapoptotic protein Bcl2 was increased. In contrast, the apoptosis rate of FLS increased and the OD values decreased due to a reduction in autophagy and proliferation, as well as increased apoptosis. The CIA rat model exhibited decreased PI3K, AKT, Beclin1, and LC3II protein expression and increased mTOR counterpart; this led to increased rates of apoptosis and reduced OD values, suggesting that FLS proliferation was inhibited. Beclin1, a homolog of ATG6, may be involved in the regulation of autophagy by activating Vps34. Moreover, the Beclin1-Vps34 complex may promote phosphatidylinositol 3-phosphate production and autophagosome maturation. Thus, Beclin1 has the potential to interact with the PI3K complex [28, 29]. LC3 is the signature protein used to detect autophagy and is the first autophagic vesicle membrane protein. Beclin1 and LC3 are located in the synovial lining of cells, and the expression of Beclin1 and LC3 is contrary to that of apoptotic markers [30, 31]. The PI3K/AKT/mTOR pathway is a significant intracellular signaling pathway regulating the cell cycle and is closely associated with cell proliferation and lifespan [32, 33]. PI3K is a heterodimer triggered by interacting with various receptors on the cell surface, such as growth factors, and changing their conformation, which is activated by phosphorylation of the intracellular second messenger 4,5-bis phosphatidylinositol. Its activation downstream of the major effector protein leads to the induction of AKT transport from the cell membrane to the cytoplasm, followed by the promotion of conformational changes and activation phosphorylation [34]. In addition, activated p-AKT regulates the promotion of cell proliferation, metastasis, and apoptosis by launching the downstream target gene mTOR [35]. Studies have already demonstrated that mTOR is involved in the negative regulation of autophagy (i.e., mTOR activation inhibits autophagy, and inhibiting mTOR induces autophagy) [36]. mTOR consists of two different functional proteins: mTORC1 and mTORC2. The former controls autophagy depending on nutrient supply and upregulates autophagy to replenish the body with nutrients when nutrients are lacking [37]. However, the association of mTORC2 with autophagy remains unclear. The Bcl2 protein family regulates the apoptosis signaling pathway. It includes the Bcl2, Bax, and BH3 subfamilies. It has been shown that Bcl2 could bind to the BH3 structural domain of Beclin1 to promote antiautophagic effects [38].

## 5. Conclusion

This study showed that miRNA-146a and autophagy are involved in regulating apoptosis and FLS proliferation. WJR treatment of a CIA rat model may regulate autophagy by influencing the miRNA-146a-mediated PI3K/AKT/mTOR pathway, thereby inhibiting apoptosis and the proliferation of FLS. This study provides a potential novel therapeutic strategy for the treatment of RA, although the role of WJR and that of miRNA-146a in other pathways in the treatment of RA may require further investigation to elucidate its mechanism and evaluate its efficacy in a clinical setting.

## Figures and Tables

**Table 1:** Primer sequences for RT-qPCR.

Gene		Sequence (5′ to 3′)
ATG5	Forward	5′-AAAGATGTGCTTCGAGATGTGT-3′
	Reverse	5′-CACTTTGTCAGTTACCAACGTCA-3′
ATG7	Forward	5′-GTGTACGATCCCTGTAACCTAACCC-3′
	Reverse	5′-CGAAAGCAGAGAACTTCAACAGACT-3′
ATG12	Forward	5′-GCCTCGGAGCAGTTGTTTA-3′
	Reverse	5′-ATGTAGGACCAGTTTACCATCAC-3′
GAPDH	Forward	5′-GGTGATGCTGGTGCTGAGTA-3′
	Reverse	5′-CGGAGATGATGACCCTTTTG-3′
U6 snRNA	Forward	5′-CTCGCTTCGGCAGCACA-3′
	Reverse	5′-AACGCTTCACGAATTTGCGT-3′
miRNA-146a		5′-TGAGAACTGAATTCCATGGGTT-3′

**Table 2:** Characteristics of chemical components in WJR by UHPLC-Q-TOF/MS analysis.

No.	Component name	Retention time	Formula	Precursor mass	Found at mass	Mass error (ppm)	Detector count	Response
1	3′-O-Angelioyl hydrellol	0.77	C_20_H_22_O_6_	358.141	359.151	1.4	1104	176
2	Ornithine	0.81	C_5_H_12_N_2_O_2_	132.089	133.097	1.4	6547	439
3	Alanine	0.82	C_3_H_7_NO_2_	89.047	90.0548	-1.6	719	636
4	Isoleucine	0.84	C_6_H_13_NO_2_	131.094	132.101	-0.3	5484	127
5	Betaine	0.92	C_5_H_11_NO_2_	117.078	118.086	-0.4	3696	3355
6	Coix lactone	1.44	C_8_H_7_NO_3_	165.042	166.0492	-4.3	778	632
7	2-Hydroxy-5-methylacetophenone	4.74	C_9_H_10_O_2_	150.068	151.0752	-1	3957	1890
8	Chlorogenic acid	5.45	C_16_H_18_O_9_	354.095	355.102	-0.2	16472	911
9	Danshensu methyl ester	5.46	C_10_H_12_O_5_	212.068	213.077	-0.2	13637	592
10	Elemisin	5.63	C_12_H_16_O_3_	208.109	209.117	-0.5	468	170
11	Luteolin	6.07	C_15_H_10_O_6_	286.047	287.055	3	760	616
12	5-Hydroxymethylfurfural	6.17	C_6_H_6_O_3_	126.031	127.039	0.5	5883	5470
13	Erythraline	6.19	C_18_H_19_NO_3_	297.136	298.144	1	9428	775
14	Ericin	6.19	C_17_H_24_O_9_	372.142	373.1489	-1.1	71733	379
15	Mesonine	6.32	C_16_H_23_NO_5_	309.157	310.164	-0.8	3788	3169
16	Phenylpropionic acid	6.88	C_9_H_11_NO_2_	165.078	166.086	-0.4	1396	274
17	Neobic Angelica ether	6.94	C_17_H_16_O_6_	316.094	317.102	0.3	33704	8608
18	Aglycone	7.18	C_14_H_14_O_4_	246.089	247.096	0.4	1083	107
19	Oxidized prehulin	7.21	C_16_H_14_O_5_	286.084	287.091	-0.3	688	520
20	Schiolaricin	7.32	C_20_H_26_O_6_	362.173	363.182	5.6	3669	1726
21	Pulsatilla	7.47	C_10_H_8_O_4_	192.042	193.049	0.4	788	703
22	Bikukuling	8.01	C_20_H_17_NO_6_	367.105	368.1131	0.8	410	125
23	3,4-Dihydroxycinnamic acid	8.04	C_9_H_8_O_4_	180.042	181.0498	1.5	677	547
24	Cimicifolin	8.06	C_16_H_18_O_6_	306.11	307.118	0.3	147038	118940
25	Coptisine	9.51	C_19_H_13_NO_4_	319.084	320.091	-0.2	2593	2062
26	d-Nandina	9.94	C_20_H_21_NO_4_	339.147	340.1541	-0.7	1352	493
27	Dehydroepapaverine	9.96	C_21_H_23_NO_4_	353.162	354.1696	-1.2	10972	8434
28	5-O-Methyl visamiol	10.24	C_16_H_18_O_5_	290.115	291.122	-1	25822	20625
29	Dihydrosanguinarine	10.24	C_20_H_15_NO_4_	333.100	334.1076	0.6	552	394
30	Tetrandine	10.58	C_21_H_21_NO_4_	351.147	352.154	-0.9	45978	10937
31	Spearmintine	10.62	C_20_H_25_NO_3_	327.183	328.1907	-0.2	560	322
32	Thymoloside	10.71	C_21_H_26_O_10_	438.152	439.1596	-0.6	24346	15557
33	Zanthoxylin	10.91	C_11_H_6_O_4_	202.026	203.033	-0.6	1016	804
34	Dehydrofumarine A	11.12	C_22_H_23_NO_4_	365.162	366.169	-1.3	57576	44710
35	Berberine	11.12	C_20_H_17_NO_4_	335.115	336.121	-3.4	2811	599
36	Honeysuckle	12.67	C_20_H_24_O_7_	376.152	377.159	-0.6	573	467
37	3'-O-Acetylchomellol	17.12	C_17_H_18_O_6_	318.11	319.116	-2.2	446	274
38	*α*-Monolinolenic esters	27.12	C_21_H_38_O_4_	354.277	355.2842	-0.2	1749	1429
39	Beta-cineol	30.49	C_15_H_26_O	222.198	223.205	-0.9	8938	359
40	Ergosterol	30.63	C_28_H_44_O	396.339	397.3435	-7.6	218	154
1	Arginine_1	0.76	C_6_H_14_N_4_O_2_	174.11168	173.1032	-6.8	2348	2163
2	Triolein	0.77	C_57_H_104_O_6_	884.78329	883.7745	-1.7	88	70
3	Mannitol	0.81	C_6_H_14_O_6_	182.07904	181.0706	-6.3	2390	1984
4	Glutamate_1	0.82	C_5_H_9_NO_4_	147.05316	146.044	-9.1	497	290
5	Ericin	0.82	C_17_H_24_O_9_	372.14203	371.1311	-9.8	1973	121
6	(2S,3S,4S,5R)-2,3,4,5,6-Pentahydroxyhexanoic acid	0.82	C_6_H_12_O_7_	196.0583	195.0501	-4.5	3188	3188
7	6′-Sinapoylspinosin	0.83	C_39_H_44_O_18_	800.25276	799.2473	2.3	100086	99
8	Sinigrin_1	1.05	C_10_H_17_NO_9_S_2_	359.034	358.026	-1.3	24244	18775
9	Chlorogenic acid	5.21	C_16_H_18_O_9_	354.09508	353.085	-5.4	536	276
10	Sinapine	6.14	C_16_H_23_NO_5_	309.15762	354.1549	-2.7	4460	446
11	Cimicifugacin	7.04	C_22_H_28_O_11_	468.16316	467.1538	0.3	5583	3944
12	Berberine hydrogenated	9.39	C_20_H_21_NO_4_	339.14706	338.1389	-2.5	68	68
13	Dehydroepapaverine	10.08	C_21_H_23_NO_4_	353.162	352.153	-6	242	183
14	5-O-Methyl visamiol	13.87	C_16_H_18_O_5_	290.11542	289.1074	-2.6	101	96
15	Loganine	26.04	C_17_H_26_O_10_	390.1526	389.1487	8.7	721	537
16	Papaveric acid_2	26.15	C_18_H_32_O_2_	280.24023	279.2321	-2.9	402	338
17	Methyl pentadecanoate	27.4	C_16_H_32_O_2_	256.24023	255.232	-2.4	10845	9167
18	Thymoloside	27.4	C_21_H_26_O_10_	438.1526	437.143	-3.6	4142	1135
19	Octadecanoic acid_2	29.83	C_18_H_36_O_2_	284.27153	283.263	-1.2	12532	10275
20	Arachic acid	31.86	C_20_H_40_O_2_	312.30283	311.295	-0.3	1315	1044
21	Taroic acid_1	33.42	C_22_H_44_O_2_	340.33413	339.3257	-3.3	4302	3443
22	Octadecanoic acid_2	33.45	C_18_H_36_O_2_	284.27153	283.2629	-4.7	2431	1651
23	Beta-carotene_2	33.8	C_35_H_60_O_6_	576.43899	575.43	-2.2	25104	794
24	Myristic acid (tetradecanoic acid)_1	34.13	C_14_H_28_O_2_	228.20893	227.2	-0.9	1811	1307
25	Methyl hexadecanoate	34.13	C_17_H_34_O_2_	270.25588	269.249	2.9	961	457
26	Palmitoleic acid	34.13	C_16_H_30_O_2_	254.22458	253.2164	-3.6	1102	933
27	Methyl myristate	34.14	C_15_H_30_O_2_	242.22458	241.2167	-2.6	1204	627
28	Di-(2-ethyl)hexyl sebacate	34.15	C_26_H_50_O_4_	426.37091	425.362	-3.5	3618	373
29	Dehydrofumarine A	34.21	C_22_H_23_NO_4_	365.162	410.163	6.2	260	258
30	Marmarin A	34.21	C_22_H_27_NO_4_	369.19401	368.1875	0.8	142	142
31	Saulatine	34.21	C_22_H_23_NO_6_	397.15254	396.1451	-0.3	253	181

## Data Availability

The data and materials used to support the findings of this study are available from the corresponding author upon request.

## References

[1] Brink M., Hansson M., Mathsson-Alm L. (2016). Rheumatoid factor isotypes in relation to antibodies against citrullinated peptides and carbamylated proteins before the onset of rheumatoid arthritis. *Arthritis Research & Therapy*.

[2] Farzana M., Kumar M. (2020). Effect of Jacobson's progressive relaxation technique over sleep disturbances and quality of life in chronic rheumatoid arthritis. *Indian Journal of Public Health Research & Development*.

[3] Atzeni F., Talotta R., Masala I. F., Bongiovanni S., Boccassini L., Sarzi-Puttini P. (2017). Biomarkers in rheumatoid arthritis. *The Israel Medical Association Journal*.

[4] van der Woude D., van der Helm-van Mil A. H. M. (2018). Update on the epidemiology, risk factors, and disease outcomes of rheumatoid arthritis. *Best Practice & Research. Clinical Rheumatology*.

[5] Giannini D., Antonucci M., Petrelli F., Bilia S., Alunno A., Puxeddu I. (2020). One year in review 2020: pathogenesis of rheumatoid arthritis. *Clinical and Experimental Rheumatology*.

[6] Miao H. B., Wang F., Lin S., Chen Z. (2022). Update on the role of extracellular vesicles in rheumatoid arthritis. *Expert Reviews in Molecular Medicine*.

[7] Buch M. H., Eyre S., McGonagle D. (2021). Persistent inflammatory and non-inflammatory mechanisms in refractory rheumatoid arthritis. *Nature Reviews Rheumatology*.

[8] Zhai K. F., Duan H., Cui C. Y. (2019). Liquiritin from Glycyrrhiza uralensis attenuating rheumatoid arthritis via reducing inflammation, suppressing angiogenesis, and inhibiting MAPK signaling pathway. *Journal of Agricultural and Food Chemistry*.

[9] Ahmadi K., Soleimani A., Soleimani Motlagh S., Baharvand Ahmadi S., Almasian M., Kiani A. A. (2020). Polymorphisms of Pre-miR-499 rs3746444 T/C and Pre-miR-146a rs2910164 C/G in the autoimmune diseases of rheumatoid arthritis and systemic lupus erythematosus in the west of Iran. *Iranian Journal of Public Health*.

[10] Zhang B., Yi J., Zhang C. L. (2017). miR-146a inhibits proliferation and induces apoptosis in murine osteoblastic MC3T3-E1 by regulating Bcl2. *European Review for Medical and Pharmacological Sciences*.

[11] Yang Z., Goronzy J. J., Weyand C. M. (2015). Autophagy in autoimmune disease. *Journal of Molecular Medicine*.

[12] Vomero M., Barbati C., Colasanti T. (2018). Autophagy and rheumatoid arthritis: current knowledges and future perspectives. *Frontiers in Immunology*.

[13] Zhu L., Wang H., Wu Y., He Z., Qin Y., Shen Q. (2017). The autophagy level is increased in the synovial tissues of patients with active rheumatoid arthritis and is correlated with disease severity. *Mediators of Inflammation*.

[14] Malemud C. J. (2013). Intracellular signaling pathways in rheumatoid arthritis. *Journal of clinical & Cellular Immunology*.

[15] Bruyn G. A., Tate G., Caeiro F. (2008). Everolimus in patients with rheumatoid arthritis receiving concomitant methotrexate: a 3-month, double-blind, randomised, placebo-controlled, parallel-group, proof-of-concept study. *Annals of the Rheumatic Diseases*.

[16] Smolen J. S., Aletaha D., Barton A., Burmester G. R., Emery P., Firestein G. S. (2018). Rheumatoid Arthritis. *Nature Reviews Disease Primers*.

[17] Zhai K. F., Duan H., Khan G. J. (2018). Salicin from Alangium chinense ameliorates rheumatoid arthritis by modulating the Nrf2-HO-1-ROS pathways. *Journal of Agricultural and Food Chemistry*.

[18] Zhai K. F., Duan H., Chen Y. (2018). Apoptosis effects of imperatorin on synoviocytes in rheumatoid arthritis through mitochondrial/caspase-mediated pathways. *Food & Function*.

[19] Jiang S. Q., Pan T., Yu J. L. (2022). Thermal and wine processing enhanced Clematidis Radix et Rhizoma ameliorate collagen II induced rheumatoid arthritis in rats. *Journal of Ethnopharmacology*.

[20] Chen Y. J., Wu J. Y., Leung W. C. (2020). An herbal formula inhibits STAT3 signaling and attenuates bone erosion in collagen-induced arthritis rats. *Phytomedicine*.

[21] Zhong H., Zhao J. (2003). Clinical application of insect drugs. *Journal of Traditional Chinese Medicine*.

[22] Zhang C., Zhang W., Shi R., Tang B., Xie S. (2019). <i>Coix lachryma-jobi</i> extract ameliorates inflammation and oxidative stress in a complete Freund's adjuvant-induced rheumatoid arthritis model. *Pharmaceutical Biology*.

[23] Liu X. D. (2019). Effects of Wenhua Juanbi recipe on the expressions of miRNA-146a and DNA methyltransferases in peripheral blood mononuclear cells of patients with rheumatoid arthritis. *China Medical Abstracts (Internal Medicine)*.

[24] Liu X. D., Wang Y. Q., Cai L., Ye L. H., Wang F., Feng Y. Y. (2017). Effect of Wenhua Juanbi Recipe () on expression of receptor activator of nuclear factor kappa B ligand, osteoprotegerin, and tumor necrosis factor receptor superfamily member 14 in rats with collagen-induced arthritis. *Chinese Journal of Integrative Medicine*.

[25] Li Y., Feng Y. F., Liu X. T. (2021). Songorine promotes cardiac mitochondrial biogenesis via Nrf2 induction during sepsis. *Redox Biology*.

[26] Sun W., Ma J., Zhao H. (2020). Resolvin D1 suppresses pannus formation via decreasing connective tissue growth factor caused by upregulation of miRNA-146a-5p in rheumatoid arthritis. *Arthritis Research & Therapy*.

[27] Shin Y. J., Han S. H., Kim D. S. (2010). Autophagy induction and CHOP under-expression promotes survival of fibroblasts from rheumatoid arthritis patients under endoplasmic reticulum stress. *Arthritis Research & Therapy*.

[28] Zhu M., Deng G., Xing C., Nie G., Wang R. F. (2020). BECN2 (beclin 2)-mediated non-canonical autophagy in innate immune signaling and tumor development. *Autophagy*.

[29] Takacs-Vellai K., Vellai T., Puoti A. (2005). Inactivation of the autophagyg *bec-1* triggers apoptotic cell death in *C. elegans*. *Current Biology*.

[30] Chen Z. K., Wang L. H. (2008). Expression of PDCD5 gene in synovium of rheumatoid arthritis. *Chin J Rheumatol*.

[31] Pap T., Franz J. K., Hummel K. M., Jeisy E., Gay R., Gay S. (2000). Activation of synovial fibroblasts in rheumatoid arthritis: lack of expression of the tumour suppressor PTEN at sites of invasive growth and destruction. *Arthritis Research*.

[32] King D., Yeomanson D., Bryant H. E. (2015). PI3King the lock: targeting the PI3K/Akt/mTOR pathway as a novel therapeutic strategy in neuroblastoma. *Journal of Pediatric Hematology/Oncology*.

[33] Wang C., Ding C., Hua Z., Chen C., Yu J. (2020). Cangfudaotan decoction alleviates insulin resistance and improves follicular development in rats with polycystic ovary syndrome via IGF-1-PI3K/Akt- Bax/Bcl-2 pathway. *Mediators of Inflammation*.

[34] Ramakrishnan V., Kimlinger T., Haug J. (2012). Anti-myeloma activity of Akt inhibition is linked to the activation status of PI3K/Akt and MEK/ERK pathway. *PLoS One*.

[35] Luan X., Zhou X., Naqvi A. (2018). MicroRNAs and immunity in periodontal health and disease. *International Journal of Oral Science*.

[36] Hardie D. G. (2015). AMPK: positive and negative regulation, and its role in whole-body energy homeostasis. *Current Opinion in Cell Biology*.

[37] Dibble C. C., Manning B. D. (2013). Signal integration by mTORC1 coordinates nutrient input with biosynthetic output. *Nature Cell Biology*.

[38] Xu H. D., Qin Z. H. (2019). Beclin 1, Bcl-2 and autophagy. *Advances in Experimental Medicine and Biology*.

